# Binding of omeprazole to protein targets identified by monoclonal antibodies

**DOI:** 10.1371/journal.pone.0239464

**Published:** 2020-09-18

**Authors:** Naw May Pearl Cartee, Michael M. Wang

**Affiliations:** 1 Department of Neurology, University of Michigan, Ann Arbor, MI, United States of America; 2 Department of Veterans Affairs, Neurology Service, VA Ann Arbor Healthcare System, Ann Arbor, MI, United States of America; 3 Molecular and Integrative Physiology, University of Michigan, Ann Arbor, MI, United States of America; University of East Anglia, UNITED KINGDOM

## Abstract

Omeprazole is the most commonly used proton pump inhibitor (PPI), a class of medications whose therapeutic mechanism of action involves formation of a disulfide linkage to cysteine residues in the H+/K+ ATPase pump on gastric secretory cells. Covalent linkage between the sole sulfur group of omeprazole and selected cysteine residues of the pump protein results in inhibition of acid secretion in the stomach, an effect that ameliorates gastroesophageal reflux and peptic ulcer disease. PPIs, though useful for specific conditions when used transiently, are associated with diverse untoward effects when used long term. The mechanisms underlying these potential off-target effects remain unclear. PPIs may, in fact, interact with non-canonical target proteins (non-pump molecules) resulting in unexpected pathophysiological effects, but few studies describe off-target PPI binding. Here, we describe successful cloning of monoclonal antibodies against protein-bound omeprazole. We developed and used monoclonal antibodies to characterize the protein target range of omeprazole, stability of omeprazole-bound proteins, and the involvement of cysteines in binding of omeprazole to targets. We demonstrate that a wide range of diverse proteins are targeted by omeprazole. Protein complexes, detected by Western blotting, are resistant to heat, detergents, and reducing agents. Reaction of omeprazole occurs with cysteine-free proteins, is not fully inhibited by cysteine alkylation, occurs at neutral pH, and induces protein multimerization. At least two other clinically used PPIs, rabeprazole and tenatoprazole, are capable of binding to proteins in a similar fashion. We conclude that omeprazole binds to multiple proteins and is capable of forming highly stable complexes that are not dependent on disulfide linkages between the drug and protein targets. Further studies made possible by these antibodies may shed light on whether PPI-protein complexes underlie off-target untoward effects of chronic PPI use.

## Introduction

Indications for proton pump inhibitor (PPI) use include gastroesophageal reflux disease, peptic ulcer disease, and dyspepsia, concerns that are arise commonly across all populations. As such, PPIs are among the most widely prescribed medications in the world [1–3].

Omeprazole, the first PPI to receive regulatory approval, was developed as an inhibitor of the gastric acid secretion [4]. At the biochemical level, omeprazole and other PPIs inhibit the H+/K+ ATPase, a pump at the mucosal cell plasma membrane, by forming a disulfide bond between the sulfur group of the drug and one of several sulfhydryl groups of the target [4–6]. This covalent linkage permanently inactivates pump function, resulting in increases in gastric pH that are responsible for therapeutic effects.

A series of additional PPIs are now used clinically worldwide. All other PPIs on the market have a similar chemical structure as omeprazole and work through the same disulfide bond-dependent molecular mechanism. Though the oldest drug of the group, omeprazole is still the most commonly used PPI.

Pharmacological characteristics of omeprazole, as the pioneer PPI, are well established. It is known to have high protein binding function with 95–98% of the drug bound to proteins in the blood [7, 8]. The principle protein targets of omeprazole in blood is albumin, though studies in bacteria suggest that the target range of protein binding is much larger [9]. Whether omeprazole binds to proteins via disulfide bonding or other means is not established.

Omeprazole and other PPIs have come under increased scrutiny because they are pervasively prescribed and used, frequently at doses that are higher than required to inhibit acid secretion and for longer periods than are clinically indicated [10–13]. Furthermore, a series of investigations has linked PPI use to a number of conditions that include cardiovascular disease [14], osteoporosis [15], C. Difficile colitis [16], community acquired pneumonia [17], and dementia [18–20], though different groups have arrived at opposite conclusions [21, 22]. The causal effect on these conditions has been debated, though a recent randomized control study suggested that only enteral infections were increased by pantoprazole administration within a three year follow-up period [23].

Because of the widespread and chronic use of PPIs and the potential consequences of off-target effects, further information is needed about 1) the range of proteins that interact with PPIs and 2) the mechanism by which PPIs interact with non-ATPase targets. In this study, we describe the development of monoclonal antibodies against omeprazole bound to proteins. Use of these reagents reveals that omeprazole and other PPIs bind avidly to a diverse range of proteins via interactions that are both dependent and independent of disulfide bonds.

## Methods

### Antigen preparation

Keyhole limpet hemocyanin (KLH) modified by omeprazole (Ome-KLH) was prepared by reduction of the target protein followed by reaction with omeprazole. KLH (10mg/mL in PBS) was mixed with TCEP-agarose (Thermo Scientific) at one volume protein to two volumes bead slurry for 60 minutes at 37˚C. After centrifugation, KLH in the supernatant was mixed with omeprazole (5mM; Acros Organics) for 4hr at room temperature. Finally, the Ome-KLH was dialyzed against PBS. (Optimization of the Ome-KLH conjugation procedure was determined by comparing reactivity of omeprazole vs vehicle treated KLH with a disulfide and infrared dye tagged oligonucleotide (IDT); drug-reacted protein was visualized by separation using non-reducing polyacrylamide gels followed by direct scanning of the gel for high molecular weight infrared complexes. The magnitude of DNA-protein conjugation was assumed to be inversely related to efficacy of omeprazole conjugation.)

### Monoclonal antibody generation

Animal studies were reviewed and approved by the Institutional Animal Care and Use Committee of GenScript, China (ANT17-003). Methods of euthanasia were consistent with the recommendations of the Panel on Euthanasia of the American Veterinary Medical Association. Antibodies were produced by a commercial vendor (Genscript) which immunized five BALB/C and five C57BL/6 mice with Ome-KLH using a standard immunization protocol. After the third injection, polyclonal antisera were sampled to ascertain responses to immunizations. This was performed by both ELISA (against Ome-KLH and KLH; see results) and by Western blotting against protein samples treated with vehicle or omeprazole (see results). Additional studies showed that polyclonal antibody binding by ELISA was inhibited by free omeprazole by -10 to 26% (average of 10%), indicated that free omeprazole did not substantially affect antibody avidity. A single BALB/C mouse that responded robustly to Ome-KLH was used to generate hybridomas using established methods of splenic cell fusion to myeloma Sp2/0 cells which ultimately yielded clones that were selected for media that exhibited reactivity to Ome-KLH but not to KLH. A total of 13 clones were further analyzed by Western blotting against Ome-KLH and KLH, and the most avid Ome-KLH reactive antibodies were used for additional studies, including screening against Ome-treated serum, lysates, and purified proteins.

### ELISA analysis

For evaluation of polyclonal sera and clones, Ome-KLH or KLH were coated at 1 μg/ml in PBS. Binding was determined using peroxidase conjugated goat anti-mouse antibodies that were quantified using a chromogenic substrate exhibiting light absorption at 450 nm.

### Protein preparation

Protein extracts were prepared from HEK293 cell cultures treated with RIPA buffer. Human serum was purchased from Sigma, and purified proteins were obtained from R&D systems except: casein, type I collagen, type IV collagen (Sigma) and vWF (Haematologic Technologies, Inc). Native proteins were treated with PPI at concentrations specified. In some cases, proteins were reduced, as indicated, prior to treatment with PPIs.

### Western blotting

Samples were boiled in sample buffer with or without reducing agents, as indicated. Western blots [24] were performed using standard methods on nitrocellulose membranes using the monoclonal antibodies followed by fluorescent anti-mouse secondary antibodies. For carboxamidomethyl-cysteine (CAM) residue detection, rabbit monoclonal antibodies 4E7 and 52H11 (manuscript in review) were used with anti-rabbit secondaries. Final detection of signal was performed on a Li-COR Odyssey imager.

## Results

### Monoclonal antibody generation

**Fig 1:**
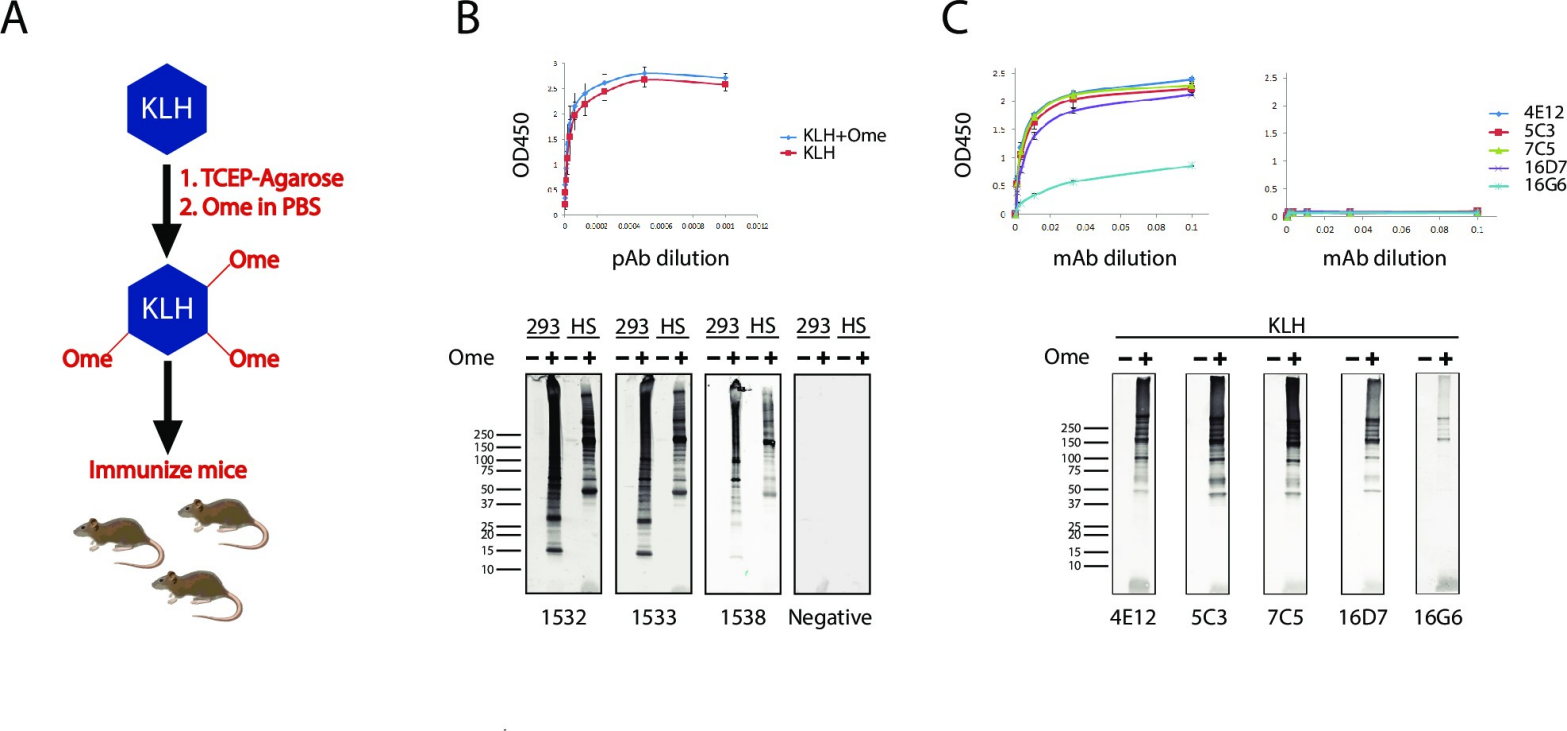
Strategy for generation of Ome-protein monoclonal antibodies. (A) KLH was reduced by TCEP agarose beads and then reacted with omeprazole to generate Ome-KLH complexes. This immunogen was then injected into 5 BALB/C and 5 C57BL/6 using a standard immunization protocol. (B) Polyclonal sera (pAb) from immunized mice contained high titers of antibodies that reacted with both KLH and Ome-KLH (ELISA results from all animals immunized; top panel); polyclonal antibodies reacted with HEK293 cell lysates (293) or human serum proteins (HS) treated with 2mM omeprazole for 2 hours at 37°C but not untreated protein. No reaction was observed for non-immune serum. (C) Monoclonal antibodies specifically recognize Ome-KLH. ELISA analysis shows five independent monoclonal antibodies that reacted with Ome-KLH (left) but not KLH alone (right). Reduced KLH was prepared as above and incubated with 2mM omeprazole for 2 hours at 37°C. Western blot analysis also indicates that monoclonal antibodies are specific for Ome-KLH (bottom panels). Samples for SDS-PAGE gel running were boiled in sample buffer without reducing agents. Antibody dilutions were 1:50 for Western blots. Molecular weight marker specifically represents the 4E12 blot.

To generate immunogenic omeprazole-protein complexes, we labeled KLH with omeprazole in a two-step process. First, we reduced KLH using TCEP-agarose beads that were then removed by centrifugation to prevent disulfide conjugates from reduction. Second, we mixed KLH and omeprazole under neutral conditions to conjugate the protein and drug. The mixture was dialyzed to remove free omeprazole, and the protein component was used to immunize mice (Fig 1A).

Polyclonal sera from immunize mice contained high levels of antibodies that reacted with KLH and Ome-KLH at the same levels; no reactivity was seen to either protein in control (non-immune) serum (Fig 1B, top panel). Western blots of proteins using polyclonal sera from immunized mice revealed reactivity against omeprazole treated cell extracts and human serum; in all animals, sera reacted with dramatically higher avidity to omeprazole treated proteins compared to vehicle treated proteins (Fig 1B, three representative animals in bottom panels), suggesting that animals were suitable for isolation of monoclonal antibodies against omeprazole-protein complexes.

After spleen cell fusion to myeloma cell lines, hybridomas were screened for production of antibodies that reacted against Ome-KLH and counter-selected against reactivity to unconjugated KLH. Multiple clones were isolated which showed the appropriate profile of antibody production (Fig 1C top panels); all of the antibodies demonstrated selectivity for omeprazole treated proteins on Western blots performed on unreduced proteins (Fig 1C bottom panels). Five representative monoclonal antibodies 4E12, 5C3, 7C5, 16D7, and 16G6 were used in all subsequent experiments.

### Monoclonal antibody binding to omeprazole treated proteins in cell lysates

**Fig 2:**
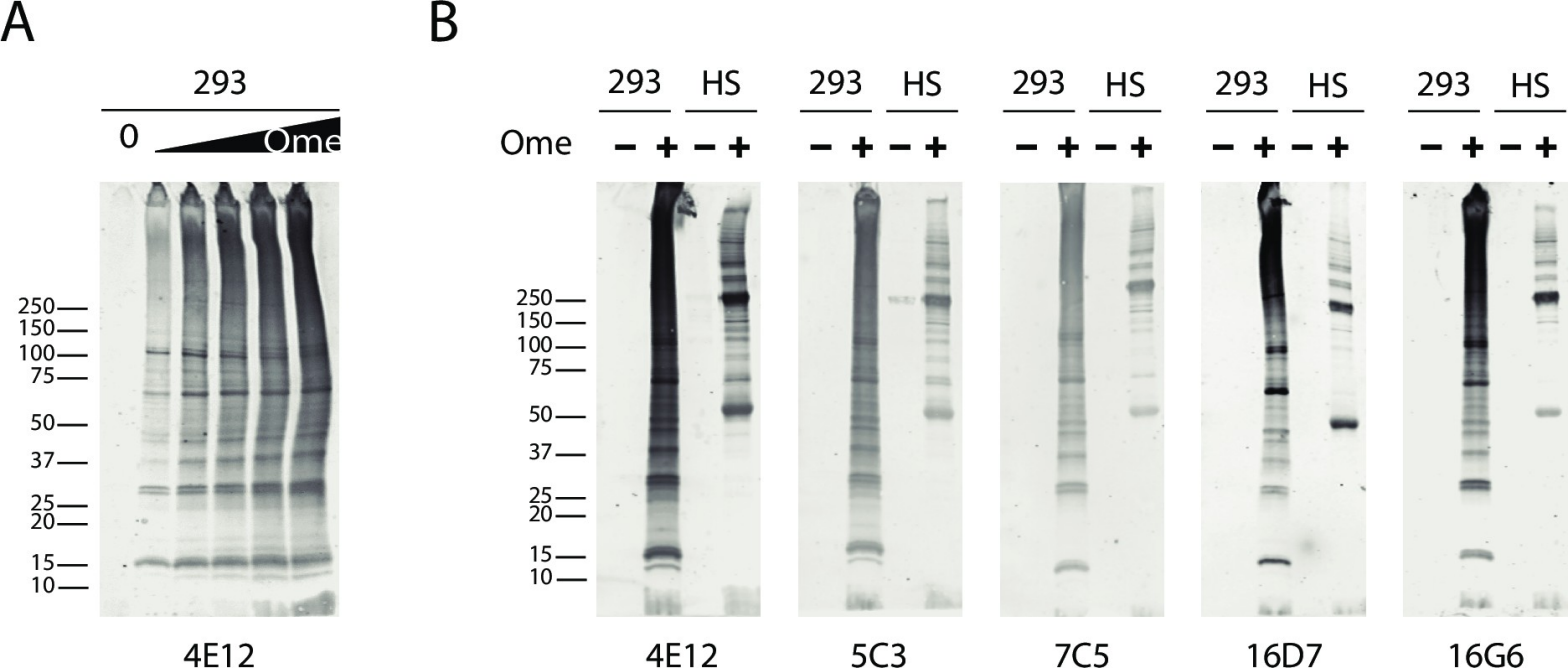
Monoclonal antibody detection of Ome-protein complexes from mammalian cells. (A) HEK293 cell lysates treated with 0, 0.1, 0.25, 0.5, 1, or 2mM omeprazole for 2 hours at 37°C indicate that the monoclonal antibody 4E12, reacts only with Ome-protein complexes and demonstrates dose dependency. (B) HEK293 lysates (293) and human serum (HS) proteins treated with 2mM omeprazole for 2hr at 37˚C also show that five monoclonal antibodies (4E12, 5C3, 7C5, 16D7, and 16G6) are specific for omeprazole bound mammalian proteins. Samples for SDS-PAGE gel running were boiled in sample buffer without reducing agents. Antibodies were used at 1:50 dilution.

To determine if omeprazole-protein (Ome-protein) epitopes are present in mammalian proteins treated with omeprazole, we used Western blotting with mAb 4E12 to examine lysates that were treated by increasing concentrations of omeprazole. Fig 2A shows a dose dependent increase in reactivity of a plethora of bands revealed by antibody 4E12 when cell lysates were treated with omeprazole. In addition, all other monoclonal antibodies tested were found to bind to multiple bands in 293 cell lysates after omeprazole treatment; no bands were detected in cell lysates that were not treated with omeprazole (Fig 2B). These studies demonstrated that omeprazole forms complexes with many proteins. Moreover, as all gels were performed using SDS, omeprazole-protein complexes are anionic detergent resistant.

### Role of disulfide bonding in omeprazole-protein complex formation

Omeprazole binds and inactivates the H+/K+ ATPase pump by formation of a reducing agent-sensitive disulfide bond to cysteine residues near the active site. An important feature of this process is that acidic pH, found in the stomach, accelerate this disulfide forming reaction. We therefore tested whether a similar mechanism of attachment was responsible for omeprazole labeling of proteins from cell extracts.

**Fig 3:**
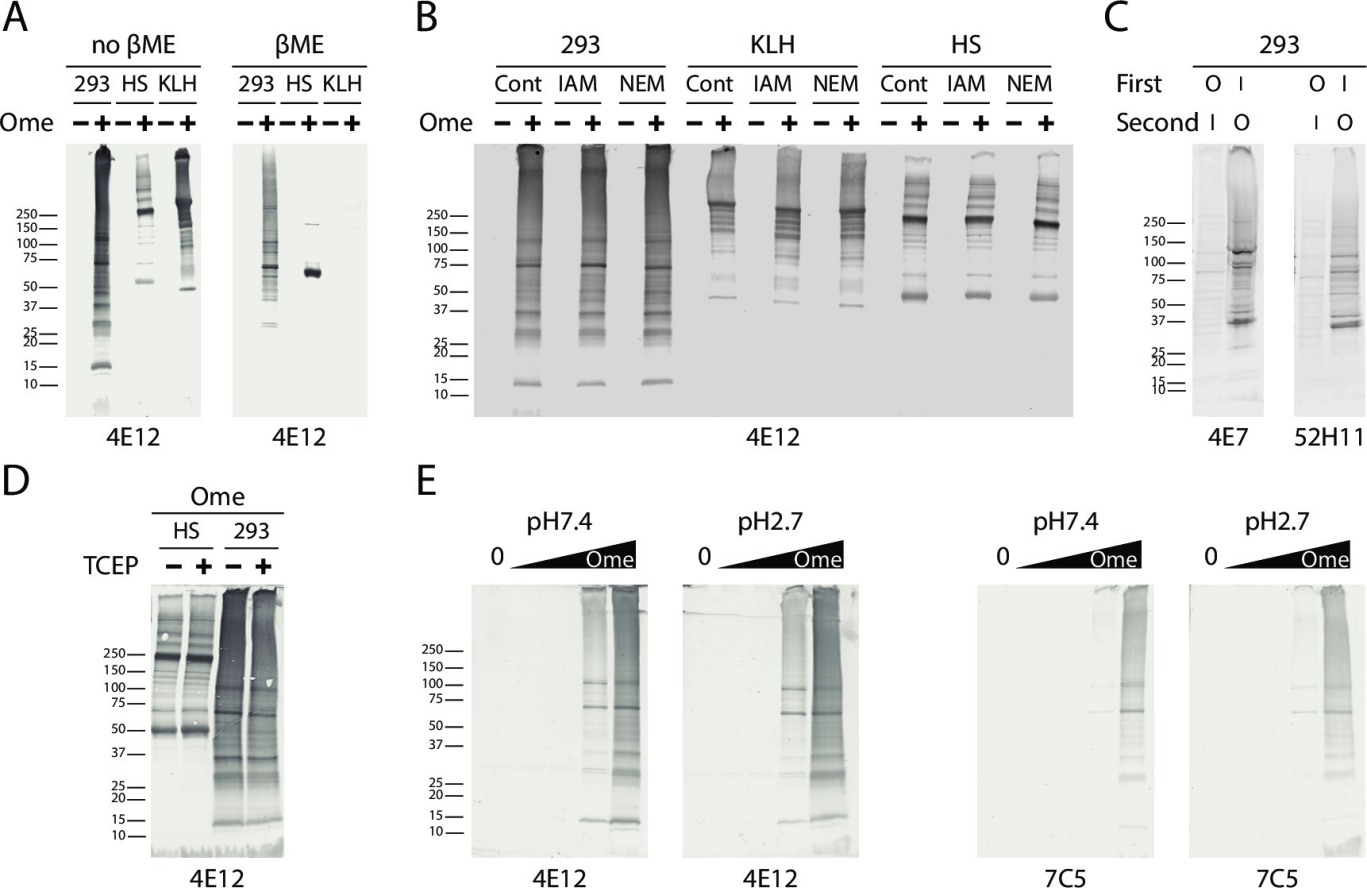
Role of redox state, cysteine residues, and pH in the formation of Ome-protein complexes. (A) Western blot analysis with 4E12 antibody, performed on non-reduced (left) and reduced (right) proteins (proteins in PBS co-incubated with 2mM omeprazole for 2 hours at 37°C), indicates that βME did not fully eliminate many of the protein omeprazole complexes. This was most noted with HEK293 lysates (293) and human serum (HS) proteins, implicating involvement of non-cysteine residues in bonding interactions. Ome-KLH, however, showed marked sensitivity to βME. Band quantification revealed 65%, 50%, and 95% of signal reduction was observed for HEK293, HS, and KLH Ome-protein complexes after treatment with βME. (B) HEK293 lysates, KLH, and HS proteins were pre-incubated with vehicle (Cont) or 10mM IAM or NEM for 2 hours at 37°C prior to reaction with 1mM omeprazole for 2 hours at 37°C. All treatments failed to block the binding of omeprazole to proteins as detected by the 4E12 antibody (non-reducing condition for SDS-PAGE) which indicate that omeprazole binding does not require cysteine sulfhydryl residues. (C) Pre-incubation of 2mM omeprazole (O) to HEK293 lysates for 2hr at 37˚C followed by 2mM IAM (I) for 2 hours at 37°C inhibited detection of carboxamidomethyl cysteine (CAM-cys) labeling of protein by antibodies, 4E7 (left) and 52H11 (right). The blockade of CAM-cys formation is consistent with omeprazole competition for sulfhydryl residues that normally react with IAM (proteins were reduced prior to SDS-PAGE). (D) Effect of pre-reduction of human serum proteins and HEK293 lysates by TCEP agarose of omeprazole labeling. TCEP pretreatment, compared with non-reduced proteins, labeled to same extent with omeprazole as shown by Westerns using 4E12 which indicates that omeprazole binding in these proteins involves interactions beyond disulfide bond formation (1mM omeprazole at 37°C for 2 hours, non-reducing SDS-PAGE). (E) Effect of pH on omeprazole labeling of protein. A dose ramp of omeprazole (0, 0.001, 0.01, 0.1, 1mM omeprazole) with HEK293 lysates was conducted at pH7.4 and 2.7 at 37˚C (2 hour co-incubation). No significant differences between the two reaction groups indicate that omeprazole is able to react similarly at neutral and acidic conditions (non-reducing SDS-PAGE). All the samples (A-E) were boiled for 3 minutes in sample buffer prior to analysis. In a separate analysis, we determined that sample boiling before addition of omeprazole reduced Ome-protein complex detection (S1 Fig).

Protein extracts reacted with 2mM omeprazole for 2 hours at 37°C were subsequently reduced with beta-mercaptoethanol before Western blot analysis to determine whether the omeprazole labeling could be reversed. There was modestly decreased binding of Ome-protein antibodies to proteins treated with reducing agents compared to non-reduced protein (~65, 50, and 95% reduction in 4E12 binding for HEK293 cell lysates, human serum and KLH, respectively; Fig 3A). As such, the Ome-protein interactions likely include both disulfide and non-disulfide interactions. The diminishment of Ome-KLH detection in SDS-PAGE after treatment with beta-mercaptoethanol is consistent with a covalent linkage of omeprazole to cysteine in a subset of drug-protein complexes.

To test if reduced cysteines in proteins participate in Ome-protein complex formation, we also pretreated proteins with cysteine alkylating agents prior to Ome-protein complex formation. Proteins pre-challenged with either N-ethylmaleimide (NEM) or iodoacetamide (IAM) followed by labeling with omeprazole were equally labeled by omeprazole, as assessed by Western blot analysis (Fig 3B). A converse reaction was performed which showed that preincubation with omeprazole blocked the labeling of sulfhydryls by IAM (Fig 3C). These experiments demonstrated that omeprazole interacting sites in proteins extend beyond NEM and IAM accessible cysteines; on the other hand, omeprazole efficiently blocked alkylation of cysteine sulfhydryl groups, providing further demonstration that omeprazole binding includes cysteine sites.

In addition, we tested if pre-reduction of protein using TCEP-agarose beads could affect the magnitude of omeprazole labeling. Both pre-reduced protein and non-reduced protein labeled with equal efficiency (Fig 3D).

To determine whether omeprazole more actively conjugates with proteins in acidic conditions, we compared labeling of cell extracts at pH 7.4 and 2.7. Western blot analysis of Ome-protein complexes did not show differences in the pattern of bands nor in the intensity of complexes recognized by monoclonal antibodies (Fig 3E). Overall, these experiments demonstrate that omeprazole binds to heterogeneous proteins via both cysteine-independent and cysteine-dependent mechanisms, and the overall magnitude of omeprazole binding to proteins is not markedly enhanced by low pH.

### Omeprazole binding to specific proteins

**Fig 4:**
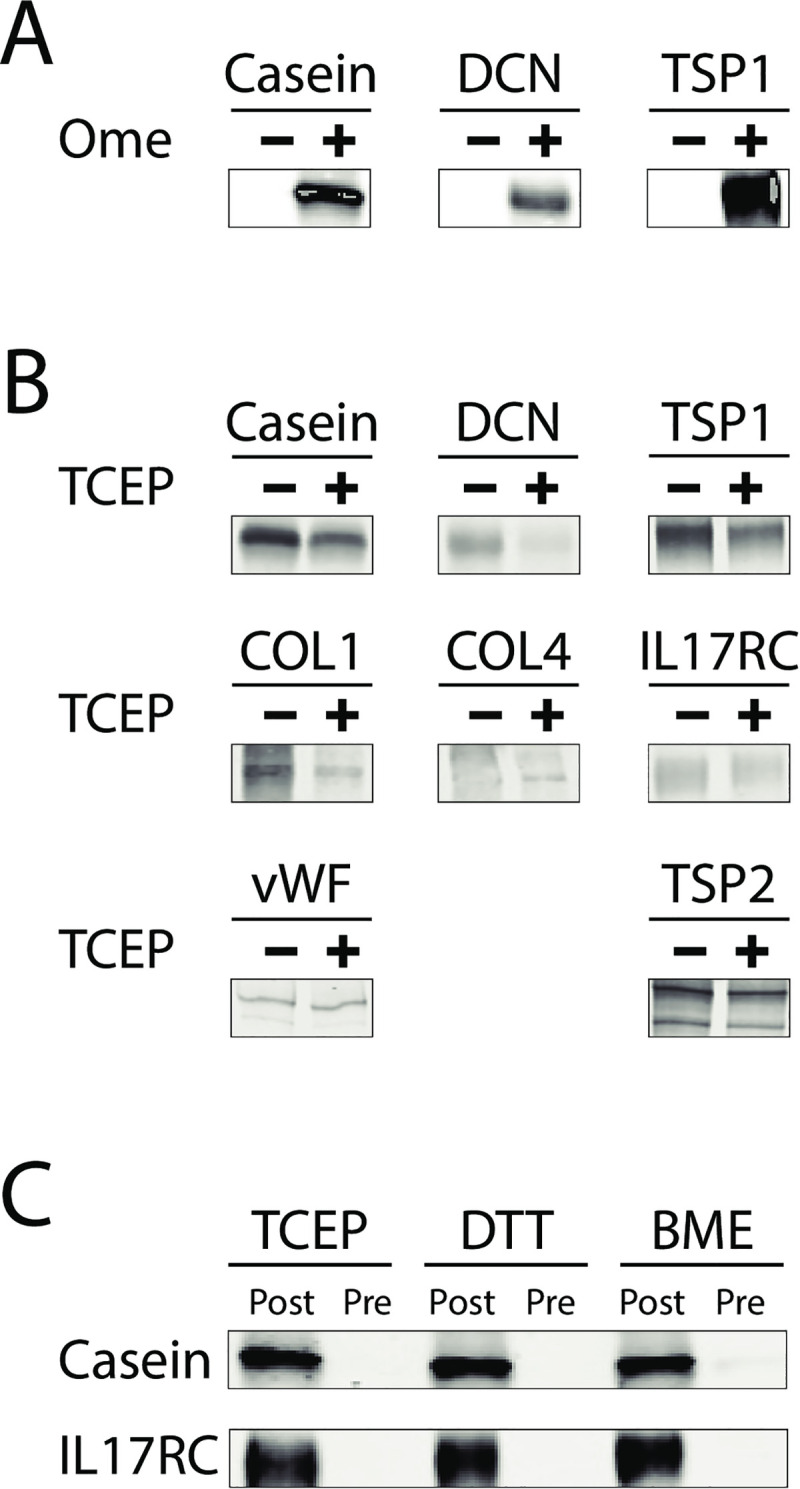
Formation of omeprazole complexes with purified proteins. (A) The ability of omeprazole to form complexes with purified proteins was tested by incubation of 1mM omeprazole with casein, DCN, and TSP1 for 2hour at 37˚C (non-reducing SDS-PAGE). Western blot analysis with monoclonal antibody indicates that the antibody binds only to the omeprazole containing complexes. (B) Pre-reduction of expanded set of purified proteins with 2.5mM TCEP beads for 1hr at 37˚C does not significantly increase the recognition by the anti-omeprazole monoclonal antibody. For all proteins tested, the formation of omeprazole-protein complexes occurs in the absence of reducing agents (non-reducing SDS-PAGE). (C) Effect of reductants on Ome-protein complex formation. All reactions were performed with 2.5mM reductants added either before or after omeprazole was mixed with proteins. When reducing agents were co-incubated with omeprazole and proteins from the initial complex formation (an individual protein was incubated with reducing agents for 30 minutes followed by co-incubation with 1mM omeprazole for 2hr at 37°C), omeprazole-protein complexes were not detected. Addition of equivalent amount of reducing agents, TCEP, DTT, or BME, after complex formation (before boiling in sample loading buffer) did not eliminate the Ome-protein complexes. All samples in (A-C) were boiled for 3 minutes in sample loading buffer. Protein molecular masses for bands shown are: casein (27kD), DCN (38kD), COL1 (>250kD), COL4 (>250kD), IL17RC (75kD), vWF (multimer at >250kD), TSP1 (multimer at >250kD), and TSP2 (multimer at >250kD).

The specific proteins targeted by omeprazole in lysates and serum could not be resolved, in part because immunoprecipitation was not feasible due to high binding of omeprazole-treated tissues to solid matrices (S2 Fig). We tested whether omeprazole could form complexes with purified proteins. As shown in Fig 4A, each protein tested was capable of forming antibody reactive complexes after incubation with omeprazole. The amount of reactivity to antibody was not significantly affected by pre-incubation with TCEP-agarose for eight of eight tested proteins (Fig 4B includes casein, DCN, COL1, COL4, IL17RC, vWF, TSP1, and TSP2), suggesting that sulfhydryl residues were not the principle target of omeprazole on proteins. Of note, casein is a cysteine-free protein, which is consistent with observations that omeprazole-protein complex formations from cell lysates (Fig 3) do not require cysteine residues.

When reducing agents were used in the presence of omeprazole, we noted that Ome-protein complexes did not form (Fig 4C). However, when Ome-protein complexes were formed in the absence of reducing agents, the conjugates were stable to subsequent treatment with three independent reducing agents.

### Time and temperature dependence of omeprazole-protein complex formation

**Fig 5:**
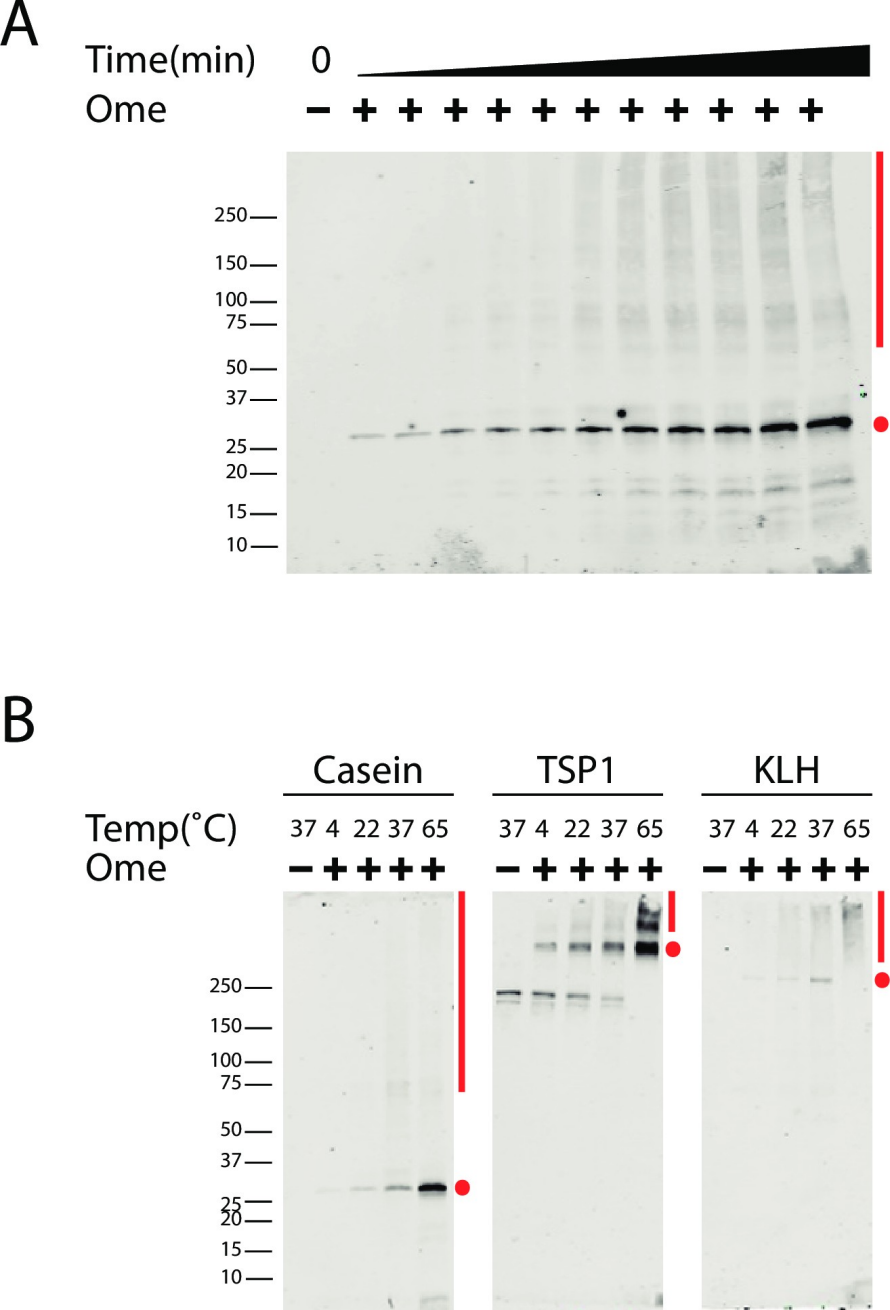
Time and temperature dependent omeprazole-protein complex formation. (A) Incubation of 1mM omeprazole with non-cysteine containing protein, casein, at 65˚C indicates a time dependent (0.5, 1, 5, 10, 15, 30, 45, 60, 75, 90, and 120 minute points) increase in omeprazole-casein complex which was recognized by the antibody. (B) Omeprazole (1mM) reaction with purified proteins, casein, TSP1, KLH, for 2hr at 4, 22, 37, and 65˚C show that there is increased complex formation with increased temperature. Note that the lowest molecular weight TSP1 band reacted with the antibody without omeprazole, representing a non-specific reaction; higher molecular weight bands are specific for omeprazole treatment. There is higher molecular weight oligomer formation with both the time and temperature dependent studies; baseline state (●) and omeprazole-dependent higher molecular weight states (**׀**) are marked. Oligomerization was especially pronounced in TSP1. There was no reducing agent in sample loading buffer, and samples were not boiled. Protein molecular masses are as follows: for casein (monomer at 27kD), TSP1 (baseline multimer >250kD), and KLH (monomer at >250kD). 5C3 antibody (1:50 dilution) was used for the detection of the samples.

To gain insight into biochemical processes that result in omeprazole interactions with proteins, we studied the effects of the PPI on casein, a protein that lacks cysteine. Over prolonged periods of time, there was a continuous increase in interactions between omeprazole and casein that did not plateau (Fig 5A).

The temperature dependence of omeprazole interactions with purified proteins was investigated in Fig 5B. Each of the proteins showed increases in complex formation with progressively elevated temperatures. Incubation at 65˚C, which is expected to result in denaturation of protein, caused the highest amount of complex formation. For all three proteins, we found that omeprazole incubation resulted in the appearance of Ome-protein species with increased apparent molecular weight. This was particularly true for TSP1, which was completely shifted to high molecular weight complexes at 65˚C.

### Ability of other PPIs to form protein complexes

**Fig 6:**
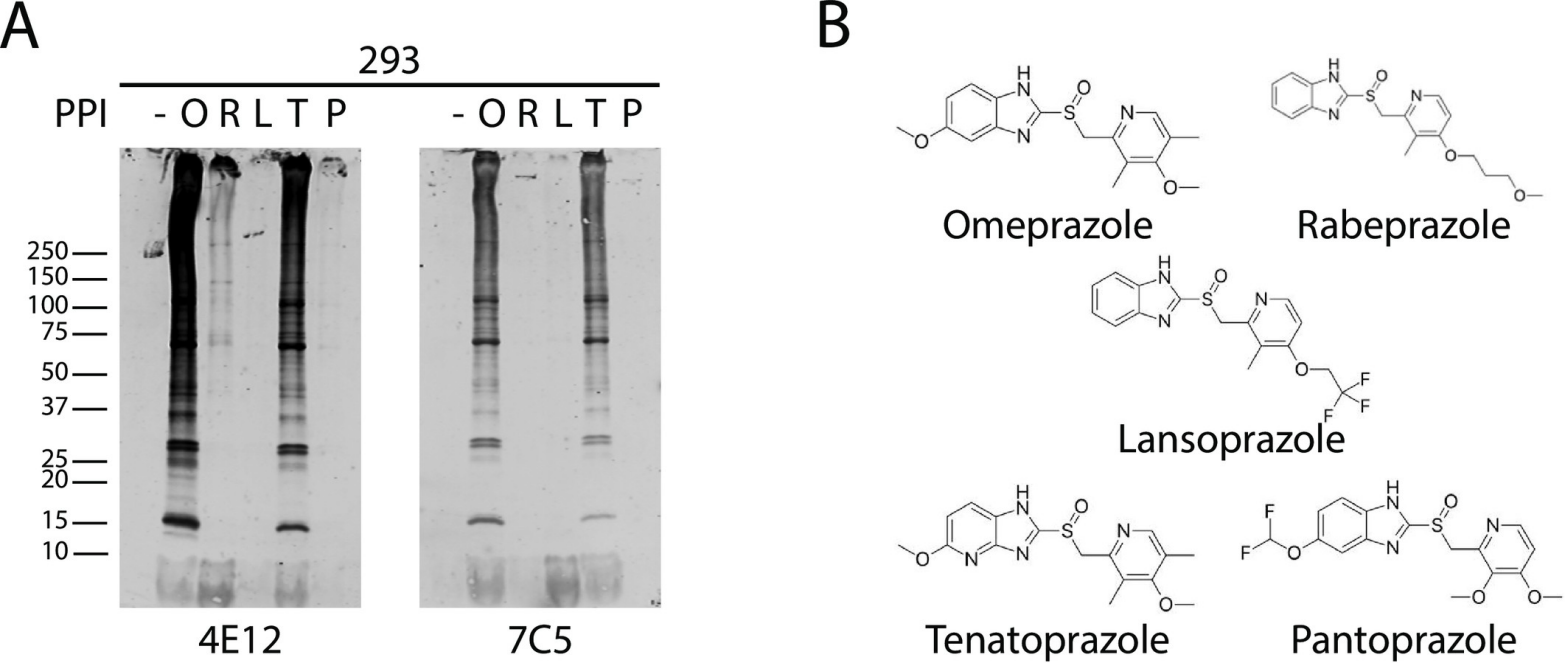
Formation of PPI-protein complexes involving tenatoprazole and rabeprazole. (A) HEK293 cell lysates treated with different PPIs, (O = Omeprazole, R = Rabeprazole, L = Lansoprazole, T = Tenatoprazole, P = Pantoprazole) 1mM at 37°C for 2 hours, shows the formation of PPI-protein complexes which were recognized by the omeprazole antibody. Tenatoprazole cross reacted the most with omeprazole antibody, followed by rabeprazole. Samples contain no reducing agent and boiled for 3 minutes. (B) A comparison of PPI structures show structural similarities of compounds tested in (A) to omeprazole.

A number of other PPIs have been developed since the introduction of omeprazole. These compounds are structurally similar to omeprazole (a substituted benzimidazole; [25]) and are thought to act in a similar mechanism via disulfide bond formation with the H+/K+ ATPase. When 293 cell lysates were treated with a set of PPIs and probed by Western blots with omeprazole-protein antibodies, we observed multiple bands in samples treated with tenatoprazole and, to a lesser degree, rabeprazole (Fig 6). Complexes were observed using at least two independent monoclonal antibodies. These studies show that monoclonal antibodies to omeprazole complexed to protein cross react with other PPI-protein complexes and demonstrate that PPIs besides omeprazole share the ability to interact with multiple proteins and to form detergent-resistant complexes.

## Discussion

The original intent of the study was to generate probes to identify sulfhydryl groups after covalent reaction with PPIs. Because PPIs react via a sulfenic acid intermediate to sulfhydryl cysteines in proton pumps [4], we reasoned that the new monoclonal antibodies could be used to map and quantify selective cysteines in their reduced state in specific proteins. However, these antibodies revealed that the range of proteins that react with PPI is broader than expected and that the mechanisms of binding extend beyond cysteine reactivity. In summary, these antibodies demonstrate two novel properties of omeprazole: 1) omeprazole appears to bind to many proteins and induces protein oligomerization; and 2) omeprazole binding to proteins occurs through both cysteine and non-cysteine dependent interactions and is largely independent of pH.

### Binding of omeprazole to multiple proteins

This studies extends observations that immunization of proteins labeled at cysteine residues can successfully generate affinity probes that can bind to proteins alkylated on thiols [26, 27]. Furthermore, the development of antibodies that recognize proteins that have been treated with omeprazole have enabled us to determine the range of PPI-targeted proteins. In cell lysates and human serum, we show that proteins of multiple molecular weights are recognized by Ome-antibodies. When purified proteins were tested, we found that every one of the molecules tested were reactive to antibodies after PPI treatment. Based on this sampling, we suggest that omeprazole binds to a large number of proteins.

The results are in agreement with the known protein binding capabilities of omeprazole; during development of the drug, it was found that it was 95% bound to serum proteins in circulation. Interaction of omeprazole with the serum protein albumin has been described and shown to depend on hydrophobic interactions [28]. Other studies have suggested targets that are independent of the proton pump. For example, omeprazole has been shown to bind reversibly to tyrosinase [29] and to inhibit its enzymatic function. Protein binding to bacteria has been proposed based on its antimicrobial activity [30] and multiple bacterial proteins were labeled in the presence of radioactive omeprazole [9]. Lansoprazole, a PPI similar to omeprazole, has been shown to have increased binding to brain in Alzheimer’s disease, a finding potentially attributable to binding to pathological tau protein [31]. The mitochondrial transporter CACT has been shown to be sensitive to omeprazole and to interact with the drug via covalent and non-covalent interactions [32]. PPIs have also been shown to affect nuclear liver X receptor function [33] and the aryl hydrocarbon receptor signaling system [34], though the effects are likely not due to irreversible protein interactions. In sum, prior work suggested modest diversity of proteins could potentially interact with PPIs. Our studies now suggest that the proteins bound by omeprazole likely extend well beyond those that have been described before. Antibodies that recognize Ome-protein complexes should facilitate future investigations into whether drug-protein interactions participate in functions previously ascribed to omeprazole.

### Omperazole interactions with proteins are both cysteine and non-cysteine dependent

The initial intent was to develop antibodies to omeprazole bound to cysteine, with the ultimate goal of developing tools to probe sulfhydryl availability. Our current characterization of protein complexes using monoclonal reagents suggest that the antibodies are not specific for omeprazole bound to proteins via cysteine disulfide bonds. In fact, our studies demonstrate that the interaction between Ome and proteins occurs through heterogeneous chemical interactions and are stable in SDS.

Interestingly, it is likely that proteins interact with omeprazole through both cysteine and non-cysteine dependent means that may vary in importance for each molecule. For example, labeling of KLH is increased significantly (data not shown) by TCEP treatment, whereas DCN, TSP1, COL1, COL4, IL17RC, vWF, and TSP2 binding (Fig 4B) is not affected significantly by TCEP pre-treatment; this suggests that KLH interacts with omeprazole predominantly by cysteines, which is consistent with experiments that show that reducing agents render Ome-KLH largely unreactive (Fig 3A) to antibodies. On the other hand, casein, a protein devoid of cysteines, reacts with omeprazole readily, and TCEP pretreatment does not increase binding. In cell lysates, omeprazole labeling of a large number of bands is unaffected by pretreatment of proteins with IAM or NEM, suggesting a multitude of cysteine-independent binding sites. Conversely, omeprazole is able to block almost all of the IAM binding sites in protein lysates, suggesting a significant number of cysteines are available for omeprazole binding.

One consequence of omeprazole modification that occurred with some proteins (especially TSP1; Fig 5) is the induction of oligomerization; it is not clear if cysteine or non-cysteine-bound drug-binding drives this process. Among several possibilities, an increase in hydrophobicity after Ome-binding may stimulate the formation of protein multimers. It is noteworthy that TSP1 normally forms disulfide linked trimers; it remains to be seen if proteins with the propensity to multimerize are particularly sensitive to omeprazole. Consistent with this observation, in the course of our studies, we also noted that Ome-protein is unusually adherent to magnetic and agarose beads, a feature that must be overcome if antibodies are to be used for proteomic studies that require immunoprecipitation (S2 Fig).

Finally, the magnitude of enhancement of omeprazole binding to protein at low pH is much more modest than what has been demonstrated for cysteine-mediated binding to the H+/K+ ATPase and for binding of omeprazole to bacterial proteins [30]. This suggests that the omeprazole reactivity we describe for proteins herewith proceeds only in part through the canonical sulfenic acid mechanism described before.

### Limitations

These studies carry limitations. Firstly, these are in vitro studies using high concentrations of omeprazole. The extent that this informs in vivo interactions between omeprazole and proteins in patients exposed to pharmacological doses of the drug need further investigation. Though we show that tenatoprazole and lansoprazole can interact with proteins in the same fashion as omeprazole, it is unclear if other PPIs tested are able to interact because these studies rely on monoclonal antibodies, which may not bind to some PPIs. Additional reagents are required to understand the extent to which all PPIs bind to proteins. Finally, we are unable to provide a concrete cysteine-independent mechanism by which PPIs bind to proteins. The basis for the non-cysteine interactions include additional rearrangements at the sulfoxide group, which is predicted to be the most reactive part of omeprazole. Non-covalent interactions such as hydrophobic interactions are also possible; if so, they would be exceptionally strong, as the complexes are resistant to heating in ionic detergents.

### Implications and future directions

Considerable concern has been raised about multisystem potential off-target effects of PPIs, which could be magnified due to the large number of patients exposed to these drugs over long periods. These studies suggest potential mechanisms for off-target effects of PPIs. With their broad range to binding via at least two different chemical mechanisms, it is conceivable that important protein targets could be modulated by this class of drugs in people. A further potential mechanism of proteinopathy by omeprazole is drug-induced protein oligomerization. The antibodies described above provide an opportunity to detect Ome-protein complexes to confirm the presence and clearance of omeprazole-modified proteins in vivo. Moreover, the antibodies could be used to map sites in proteins that may be susceptible to PPI modification.

## Supporting information

S1 Fig Immunoblot analysis of dose dependent binding of omeprazole with native or denatured HEK293 cell lysates. HEK293 cell lysates (sonicated in RIPA buffer) diluted in PBS and incubated with 0, 1, 10, 25, 50, and 100μM omeprazole for 22 hours at 37°C. SDS-containing non-reducing sample buffer was added to the sample which was boiled for 3 minutes (A-B; left 6 lanes, Boiled after Ome). An equivalent amount of HEK293 cell lysates was added to PBS and mixed with the same amount of SDS containing non-reducing sample buffer and boiled for 3 minutes first. After cooling down to room temperature, 0, 1, 10, 25, 50, and 100μM omeprazole was added and co-incubated for 22 hours at 37°C (A-B, right 6 lanes, Boiled before Ome). (A) Immunoblotting with omeprazole monoclonal antibody 4E12 (purified antibody; 1:1000 dilution) shows dose- dependent protein-omeprazole conjugate formation. (B) LI-COR REVERT 700 total protein stain was used to visualize the total amount of protein present on the same membrane. (C) Bar graph showing a comparison of the 4E12 signal for native (black bars) and denatured (white bars) protein binding with omeprazole normalized to REVERT signal. Detectable signal starts at 10uM and denatured protein binds less omeprazole at all concentrations.
(PDF)

S2 Fig Subjection of protein-omeprazole conjugates to immunoprecipitation. HEK293 cells lysate was incubated with 5mM omeprazole for 1hr at 37°C. To remove excess omeprazole, the sample was then dialyzed in Slide-A-Lyzer Mini dialysis device (10K MWCO, Thermo Fisher) for 20 to 24 hours at 4°C. Dialyzed sample was then incubated with either PBS or 2ug purified omeprazole monoclonal antibodies (4E12 or 5C3) for 18 to 20hr at 4°C. Protein A beads (Promega) were used for immunoprecipitation of omeprazole conjugated protein. Antibody alone without HEK293 cells lysate conjugated with omeprazole was also included as a control. Multiple proteins from 293 lysates co-purify with beads in the absence of antibodies, consistent with non-specific binding of these Ome-treated proteins to protein A beads. Two protein bands were observed around 20 and 50kD in the sample IP by 5C3 (red dots) which were not present in the sample without antibody and may represent proteins that come down with 5C3. SDS-PAGE was carried out after boiling the samples for three minutes in reducing sample buffer.
(PDF)

S1 File (PDF)
