# Supplementary material for: Binding of omeprazole to protein targets identified by monoclonal antibodies
Source: PLoS One. 2020 Sep 18;15(9):e0239464. doi: 10.1371/journal.pone.0239464 (PMC7500594; doi:10.1371/journal.pone.0239464)
Supplement: S1 Fig — HEK293 cell lysates (sonicated in RIPA buffer) diluted in PBS and incubated with 0, 1, 10, 25, 50, and 100μM omeprazole for 22 hours at 37°C. SDS-containing non-reducing sample buffer was added to the sample which was boiled for 3 minutes (A-B; left 6 lanes, Boiled after Ome). An equivalent amount of HEK293 cell lysates was added to PBS and mixed with the same amount of SDS containing non-reducing sample buffer and boiled for 3 minutes first. After cooling down to room temperature, 0, 1, 10, 25, 50, and 100μM omeprazole was added and co-incubated for 22 hours at 37°C (A-B, right 6 lanes, Boiled before Ome). (A) Immunoblotting with omeprazole monoclonal antibody 4E12 (purified antibody; 1:1000 dilution) shows dose- dependent protein-omeprazole conjugate formation. (B) LI-COR REVERT 700 total protein stain was used to visualize the total amount of protein present on the same membrane. (C) Bar graph showing a comparison of the 4E12 signal for native (black bars) and denatured (white bars) protein binding with omeprazole normalized to REVERT signal. Detectable signal starts at 10uM and denatured protein binds less omeprazole at all concentrations. (PDF) [file pone.0239464.s001.pdf]

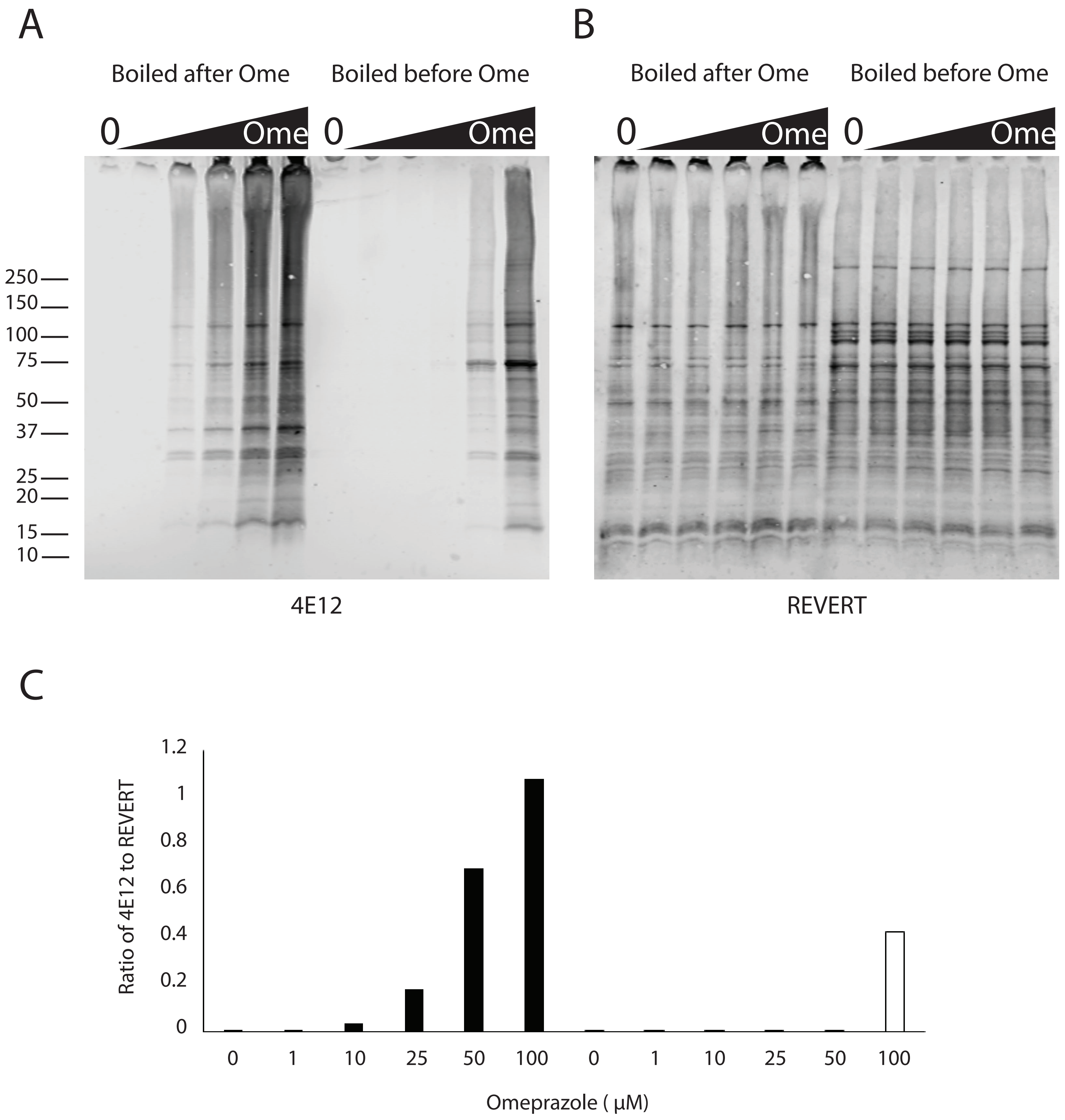

Supplemental Figure 1. Immunoblot analysis of dose dependent binding of omeprazole with native or denatured HEK293 cell lysates. HEK293 cell lysates (sonicated in RIPA buffer) diluted in PBS and incubated with 0, 1, 10, 25, 50, and 100μM omeprazole for 22 hours at 37°C. SDS-containing non-reducing sample buffer was added to the sample which was boiled for 3 minutes (A-B; left 6 lanes, Boiled after Ome). An equivalent amount of HEK293 cell lysates was added to PBS and mixed with the same amount of SDS containing non-reducing sample buffer and boiled for 3 minutes first. After cooling down to room temperature, 0, 1, 10, 25, 50, and 100μM omeprazole was added and co-incubated for 22 hours at 37°C (A-B, right 6 lanes, Boiled before Ome). (A) Immunoblotting with omeprazole monoclonal antibody 4E12 (purified antibody; 1:1000 dilution) shows dose- dependent protein-omeprazole conjugate formation. (B) LI-COR REVERT 700 total protein stain was used to visualize the total amount of protein present on the same membrane. (C) Bar graph showing a comparison of the 4E12 signal for native (black bars) and denatured (white bars) protein binding with omeprazole normalized to REVERT signal. Detectable signal starts at 10uM and denatured protein binds less omeprazole at all concentrations.
