# Supplementary material for: Binding of omeprazole to protein targets identified by monoclonal antibodies
Source: PLoS One. 2020 Sep 18;15(9):e0239464. doi: 10.1371/journal.pone.0239464 (PMC7500594; doi:10.1371/journal.pone.0239464)
Supplement: S2 Fig — HEK293 cells lysate was incubated with 5mM omeprazole for 1hr at 37°C. To remove excess omeprazole, the sample was then dialyzed in Slide-A-Lyzer Mini dialysis device (10K MWCO, Thermo Fisher) for 20 to 24 hours at 4°C. Dialyzed sample was then incubated with either PBS or 2ug purified omeprazole monoclonal antibodies (4E12 or 5C3) for 18 to 20hr at 4°C. Protein A beads (Promega) were used for immunoprecipitation of omeprazole conjugated protein. Antibody alone without HEK293 cells lysate conjugated with omeprazole was also included as a control. Multiple proteins from 293 lysates co-purify with beads in the absence of antibodies, consistent with non-specific binding of these Ome-treated proteins to protein A beads. Two protein bands were observed around 20 and 50kD in the sample IP by 5C3 (red dots) which were not present in the sample without antibody and may represent proteins that come down with 5C3. SDS-PAGE was carried out after boiling the samples for three minutes in reducing sample buffer. (PDF) [file pone.0239464.s002.pdf]

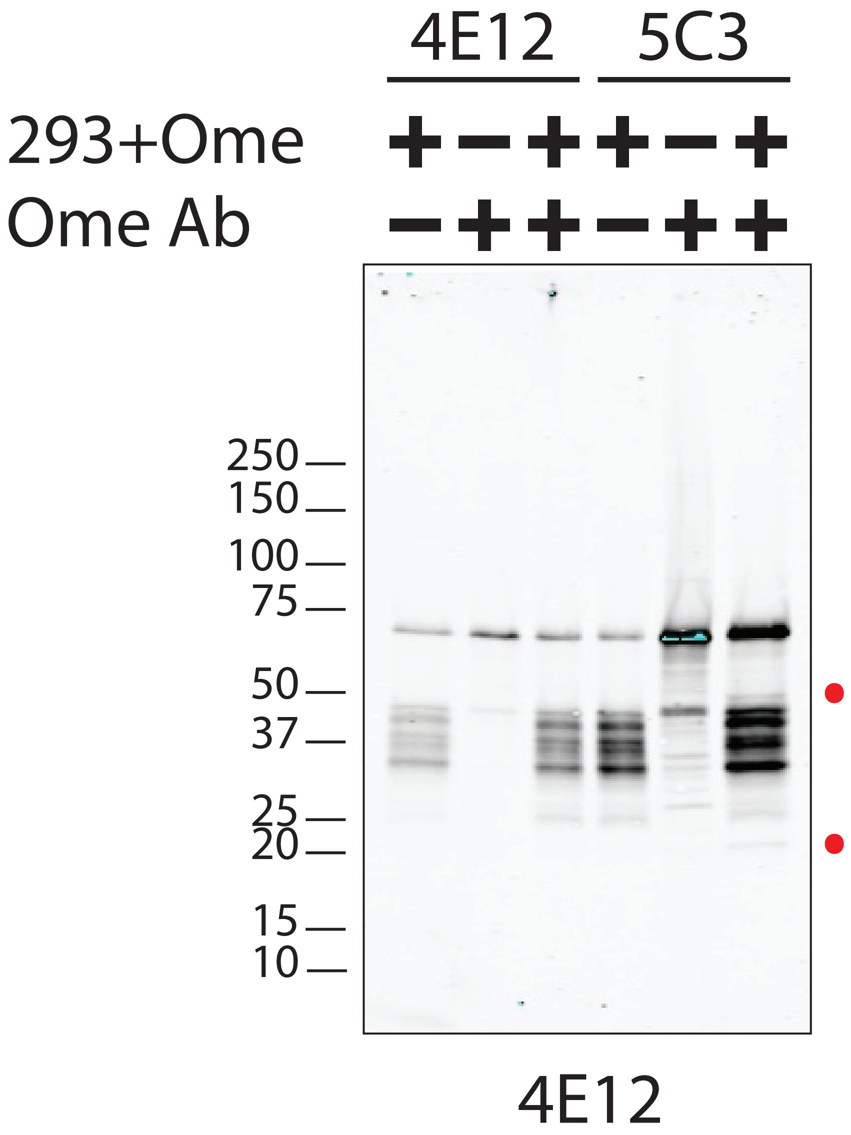

Supplemental Figure 2. Subjection of protein-omeprazole conjugates to immunoprecipitation. HEK293 cells lysate was incubated with 5mM omeprazole for 1hr at 37°C. To remove excess omeprazole, the sample was then dialyzed in Slide-A-Lyzer Mini dialysis device (10K MWCO, Thermo Fisher) for 20 to 24 hours at 4°C. Dialyzed sample was then incubated with either PBS or 2ug purified omeprazole monoclonal antibodies (4E12 or 5C3) for 18 to 20hr at 4°C. Protein A beads (Promega) were used for immunoprecipitation of omeprazole conjugated protein. Antibody alone without HEK293 cells lysate conjugated with omeprazole was also included as a control. Multiple proteins from 293 lysates co-purify with beads in the absence of antibodies, consistent with non-specific binding of these Ome-treated proteins to protein A beads. Two protein bands were observed around 20 and 50kD in the sample IP by 5C3 (red dots) which were not present in the sample without antibody and may represent proteins that come down with 5C3. SDS-PAGE was carried out after boiling the samples for three minutes in reducing sample buffer.
