# Supplementary material for: Binding of omeprazole to protein targets identified by monoclonal antibodies
Source: PLoS One. 2020 Sep 18;15(9):e0239464. doi: 10.1371/journal.pone.0239464 (PMC7500594; doi:10.1371/journal.pone.0239464)
Supplement: S1 File — (PDF) [file pone.0239464.s003.pdf]

Figure 1B

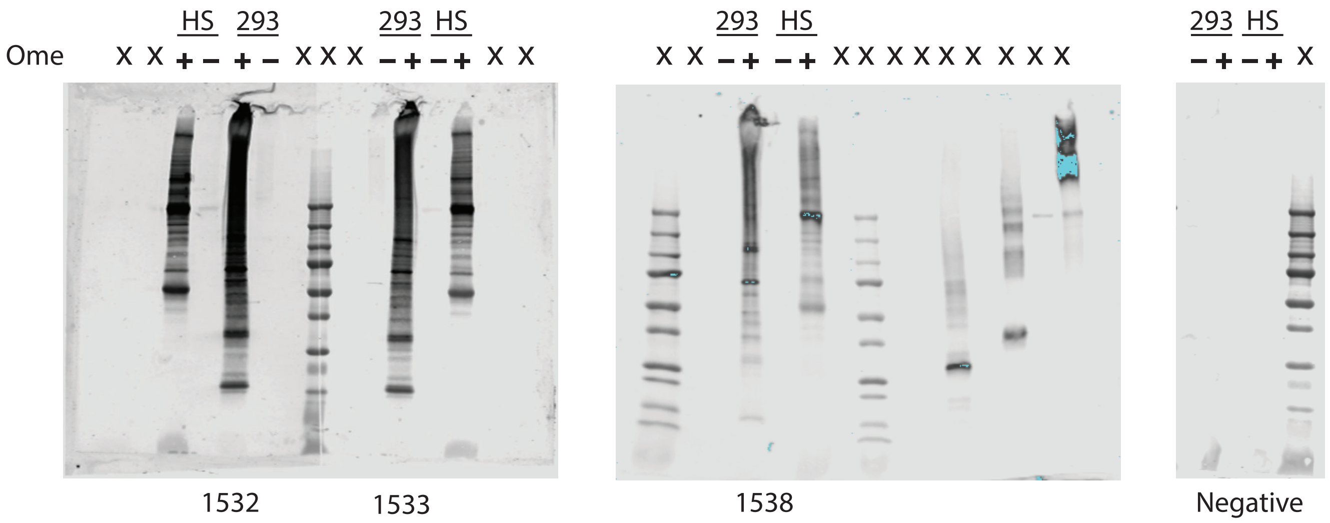

Figure 1C

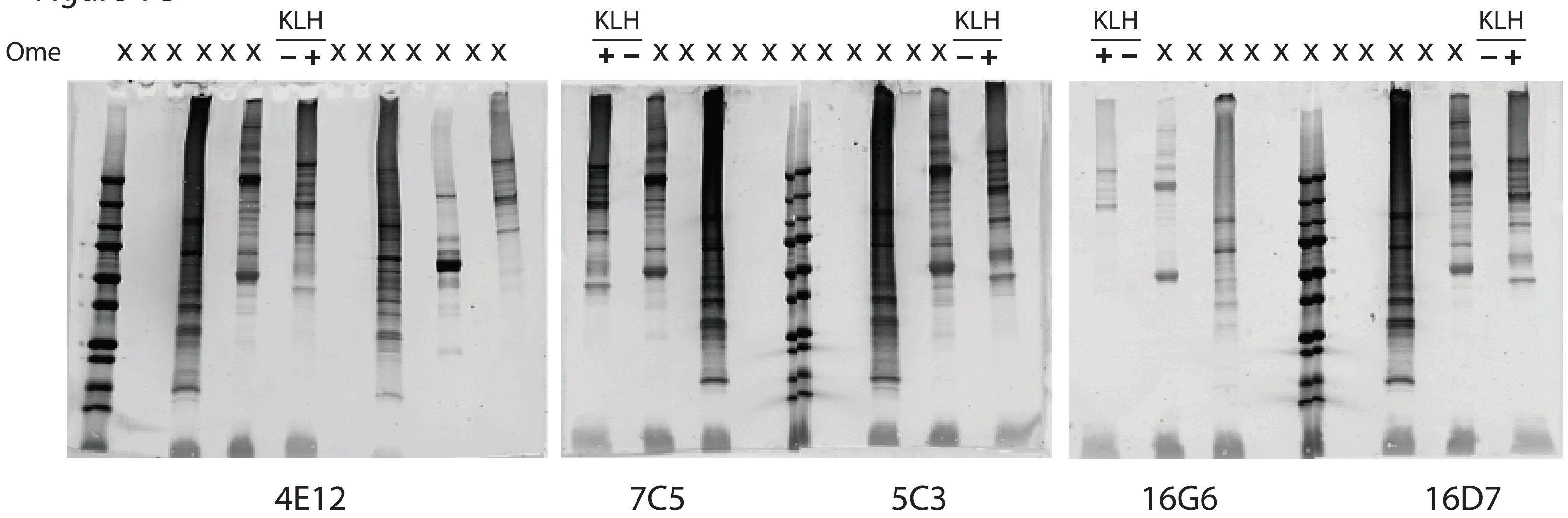

Sample wells were loaded in the indicated manner and Odyssey LICOR imager was used for the image capture.
Ome=Omeprazole
Figure 1B, 293 = HEK 293 cells lysate, HS = Human Serum
Figure 1C, KLH = Keyhole Lympet Hemocyanin

Figure 2A

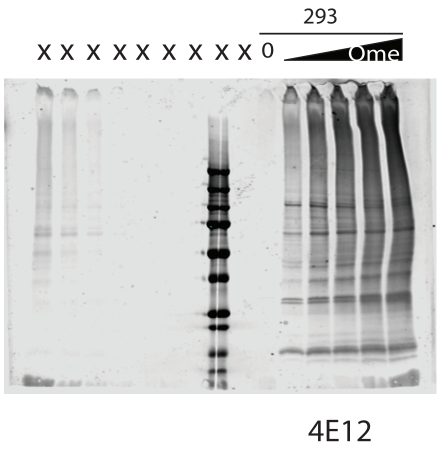

Figure 2B

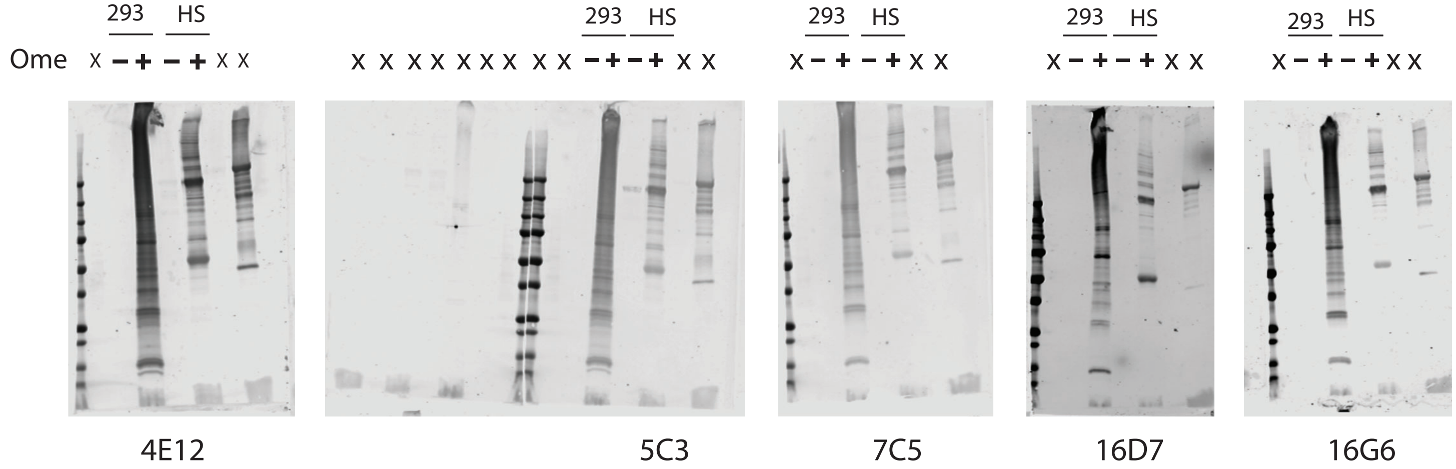

Sample wells were loaded in the indicated manner and Odyssey LICOR imager was used for the image capture.
Ome = omeprazole treatment dose in figure 1A, 0, 0.1, 0.25, 0.5, 1, 2mM. 293 = HEK 293 cells lysate,
Figure 2B, HS = Human Serum
X = sample not included in the manuscript

Figure 3A

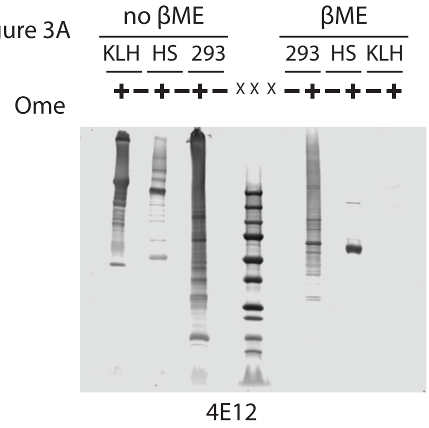

Figure 3B

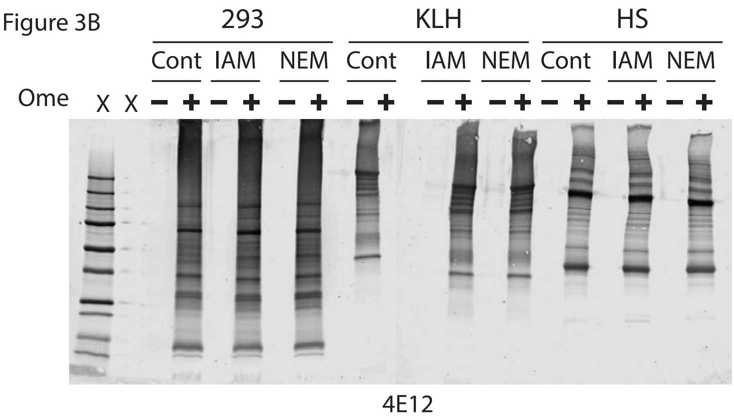

Figure 3C

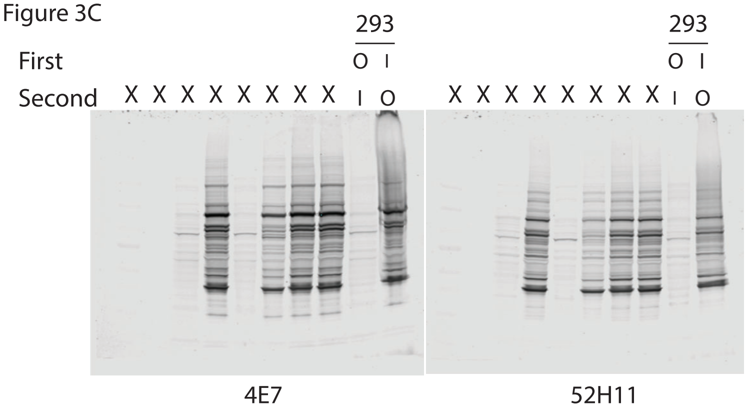

Figure 3D

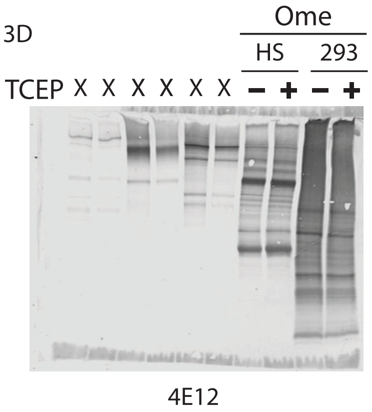

Figure 3E

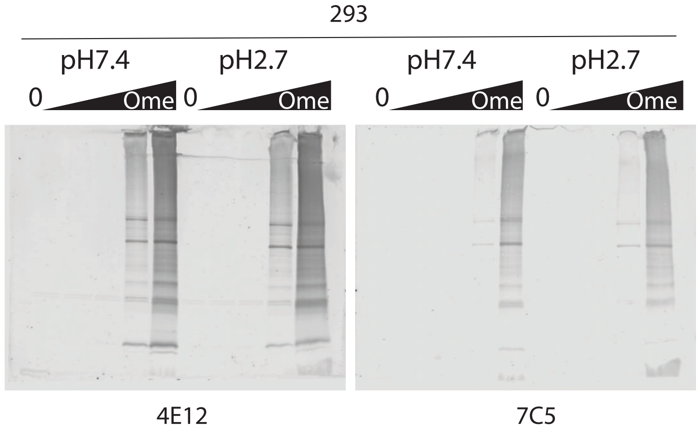

Samples wells were loaded as indicated in the figures above and Odyssey LICOR was used for the image capture.
Ome = Omeprazole, 293 = 293 cells lysate, HS = Human Serum, KLH = Keyhole Lympet Hemocyanin, X = samples not included in the manuscript figures
Figure 3B, Cont= Control, IAM = Iodoacetamide, NEM = N-ethylmaleimide
Figure 3C, I = Iodoacetamide, O = Omeprazole

Figure 4A

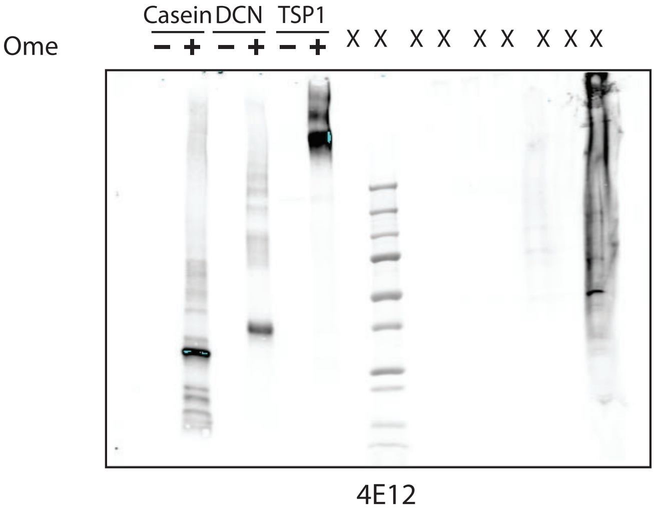

Figure 4B

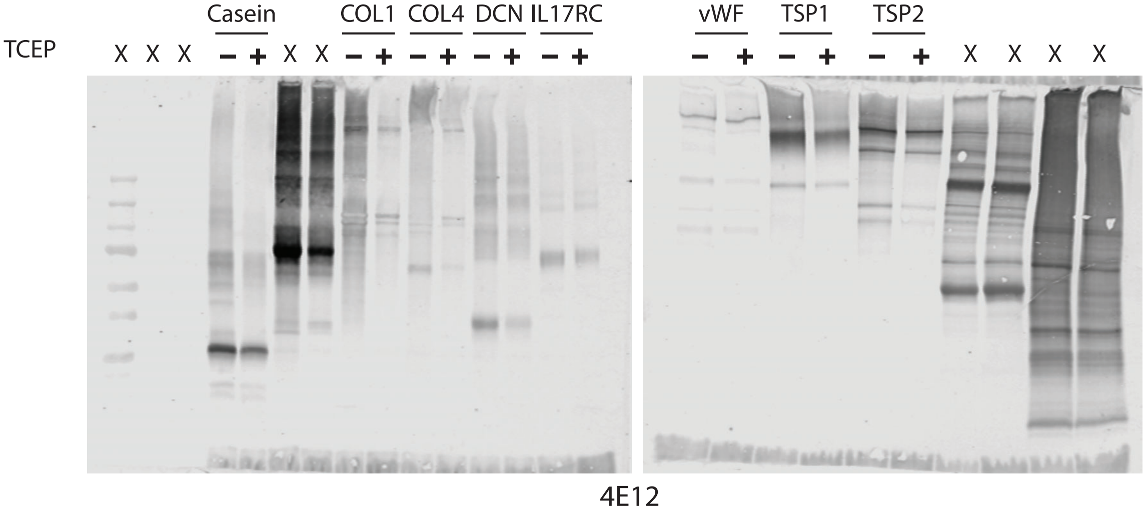

Figure 4C

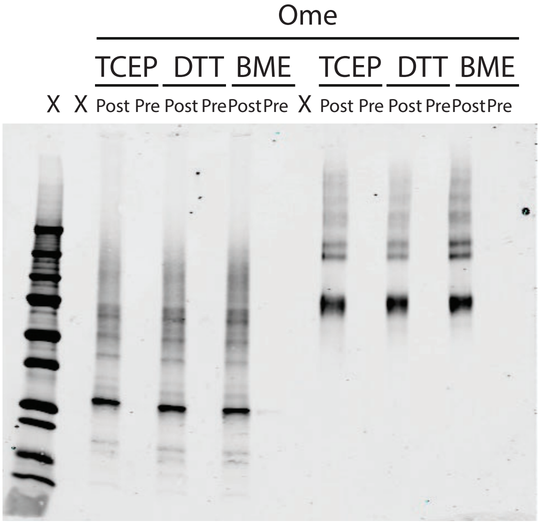

Sample wells loading are as shown in the figures. Odyssey LICOR imager was used for the image capture.

Ome = Omeprazole

X = Sample not included in the manuscript

Figure 5A

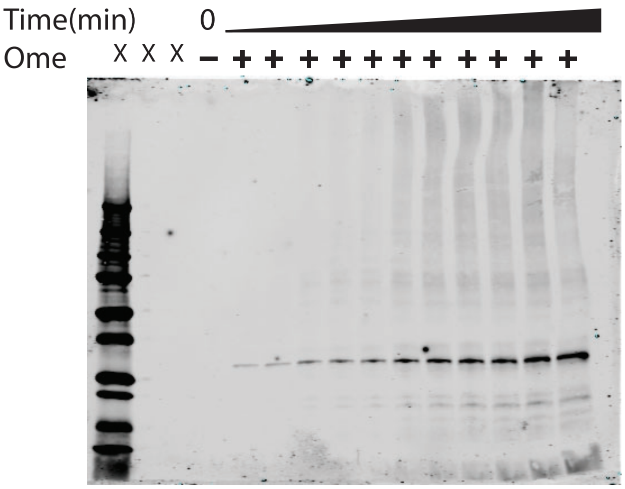

Figure 5B

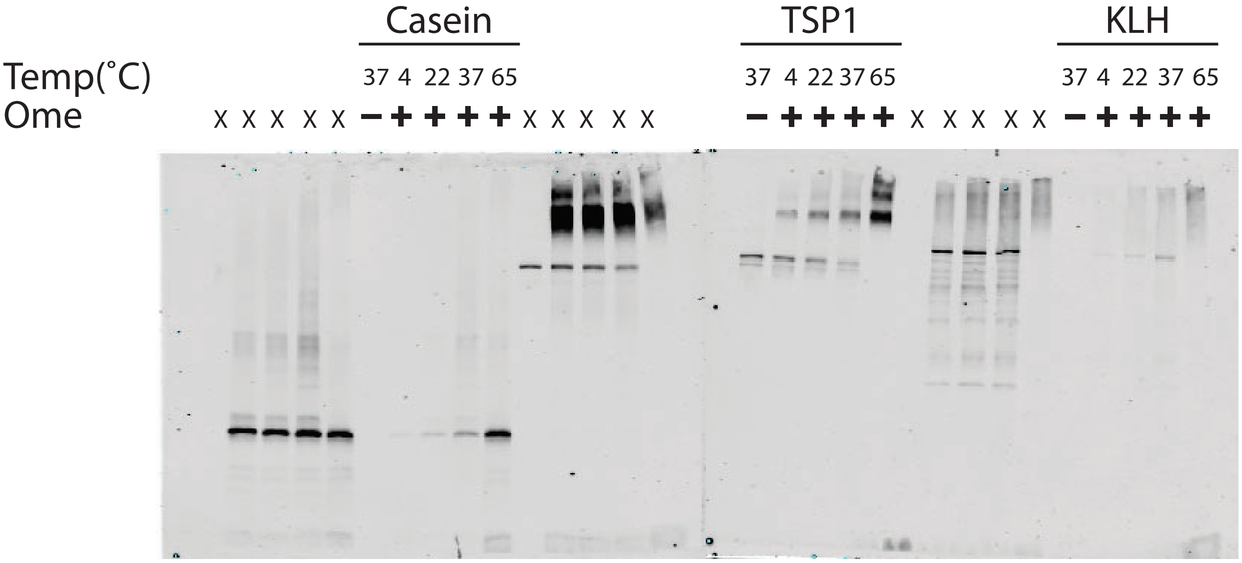

All samples were loaded as indicated and Odyssey LICOR was used for the image capture.
X = sample not included in the manuscript figures.

Figure 5A

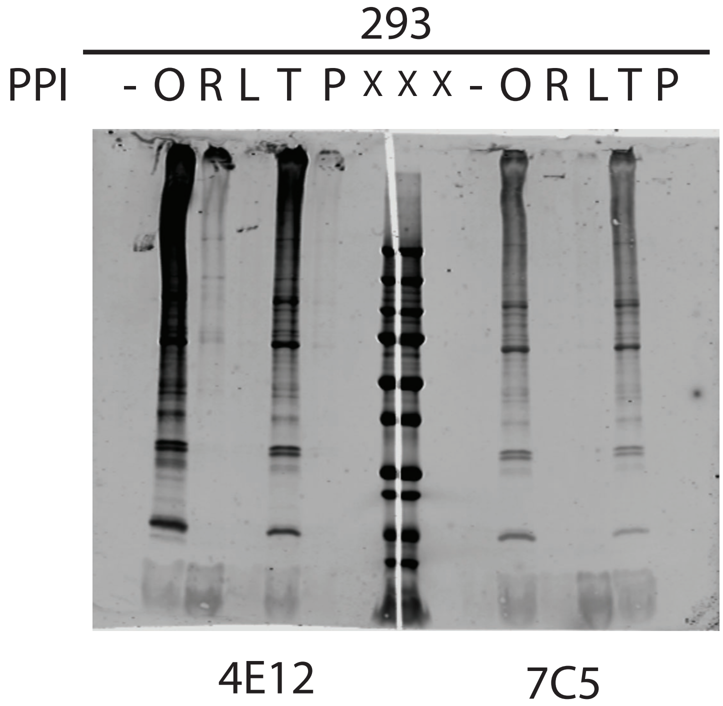

Sample wells were loaded as indicated and Odyssey LICOR imager was used for image capture.
293 = HEK 293 cells lysate
PPI = proton pump inhibitor
O = omeprazole, R = Rabeprazole, L= Lansoprazole, T = Tenatoprazole
P = Pantoprazole

Supplymental Figure A B

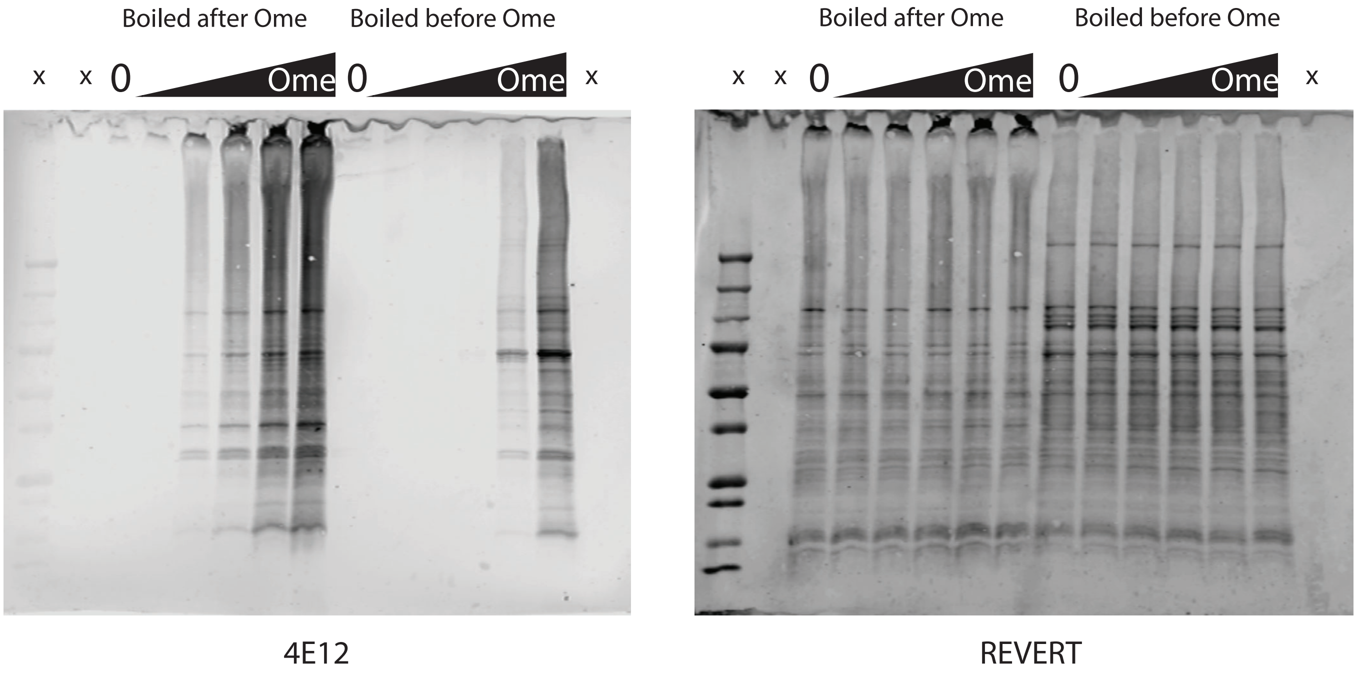

Sample wells were loaded as indicated and Odyssey LICOR imager was used for image capture.

Ome = Omeprazole concentration 1, 10, 25, 50, 100 µM.

Supplemental Figure 2

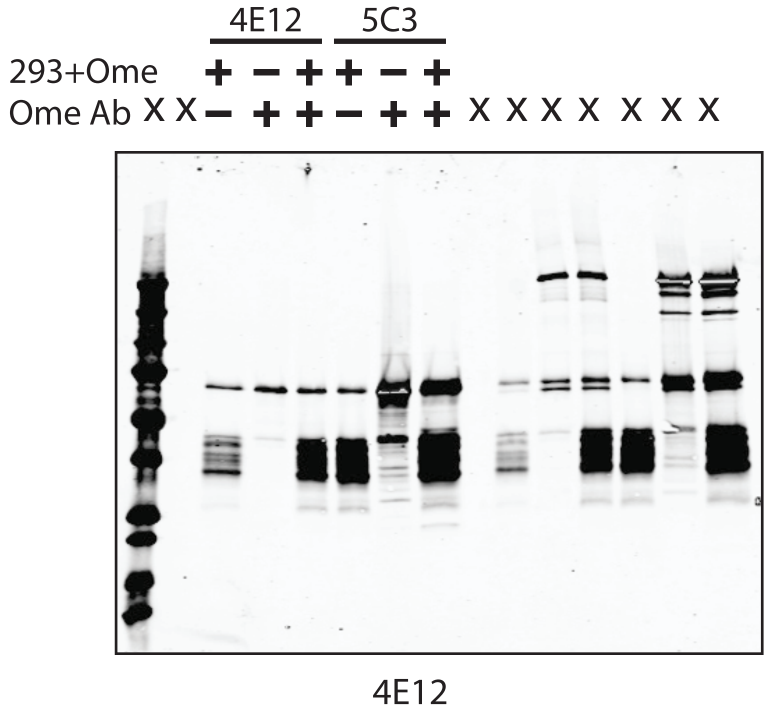

Sample wells were loaded as indicated and Odyssey LICOR imager was used for image capture.
293 = HEK 293 lysates, Ome = Omeprazole,
Ome Ab = Omeprazole monoclonal antibody
X = sample not included in the manuscript figure.
